# Occupational risk factors have to be considered in the definition of high-risk lung cancer populations

**DOI:** 10.1038/bjc.2012.75

**Published:** 2012-03-27

**Authors:** P Wild, M Gonzalez, E Bourgkard, N Courouble, C Clément-Duchêne, Y Martinet, J Févotte, C Paris

**Affiliations:** 1 INRS, rue du Morvan CS 60027, 54519, Vandoeuvre les Nancy, Cedex, France; 2 Department of Occupational Diseases 1, Hopitaux Universitaire de Strasbourg, place de l’Hôpital, 67091 Strasbourg, Cedex, France; 3 Inserm U954, 9 rue de la Forêt de Haye, 54505 Vandoeuvre les Nancy, France; 4 Department of Respiratory Medicine, CHU Nancy, rue du Morvan, 54511 Vandoeuvre les Nancy, France; 5 Unité mixte de recherche épidémiologique et de surveillance en transport, travail et environnement - Umrestte (UCB Lyon 1/Inrets), Université Claude Bernard Lyon 1 8, avenue Rockefeller 69373, Lyon, Cedex 08, France; 6 Department of Occupational Medicine, CHU Nancy, rue du Morvan, 54511 Vandoeuvre les Nancy, France

**Keywords:** lung cancer, case–control, occupational, attributable fractions

## Abstract

**Background:**

The aim of this study was to compute attributable fractions (AF) to occupational factors in an area in North-Eastern France with high lung cancer rates and a past of mining and steel industry.

**Methods:**

A population-based case–control study among males aged 40–79 was conducted, including confirmed primary lung cancer cases from all hospitals of the study region. Controls were stratified by broad age-classes, district and socioeconomic classes. Detailed occupational and personal risk factors were obtained in face-to-face interviews. Cumulative occupational exposure indices were obtained from the questionnaires. Attributable fractions were computed from multiple unconditional logistic regression models.

**Results:**

A total of 246 cases and 531 controls were included. The odds ratios (ORs) adjusted on cumulative smoking and family history of lung cancer increased significantly with the cumulative exposure indices to asbestos, polycyclic aromatic hydrocarbons and crystalline silica, and with exposure to diesel motor exhaust. The AF for occupational factors exceeded 50%, the most important contributor being crystalline silica and asbestos.

**Conclusion:**

These AFs are higher than most published figures. This can be because of the highly industrialised area or methods for exposure assessments. Occupational factors are important risk factors and should not be forgotten when defining high-risk lung cancer populations.

For several decades, numerous epidemiological studies have identified smoking and occupational carcinogen exposure such as asbestos as leading causes of lung cancers (Blot and Fraumeni, 1996). Reported fractions of lung cancer attributable to smoking, that is the fraction of avoidable lung cancer had the exposure been absent, were usually reported to be as high as 90% in males and 60% in females (Sun, Schiller and Gazdar, 2007). With respect to occupational exposures, these attributable fractions (AF) vary considerably from 6 to 17% in the US males (Steenland *et al*, 2003) or 4% in European non-smokers (Pohlabeln *et al*, 2000) up to 40% in a Swedish study (Damber and Larsson, 1985), with intermediate values in national studies in France (Imbernon, 2003) and the United Kingdom (Rushton *et al*, 2010). A recent review of these AF (De Matteis *et al*, 2008) shows that these estimates mainly depend on the industrial setting of the study area. This implies that national summaries may underestimate AFs in areas with a high prevalence of hazardous industries. This review shows also that AFs depend on the method by which the exposure is assessed with higher AFs reported when the exposure is based on expert assessment and job-exposure matrices than those based on lists of high-risk occupations. As a conclusion from this review, one can tentatively assume that a better precision of the occupational exposure assessment leads to higher AFs. Moreover, most of the published studies did not assess independently exposures to major occupational carcinogens such as asbestos, silica or polycyclic aromatic hydrocarbons (PAH).

The aim of this study was to estimate the fraction of lung cancers attributable to occupational exposures and specifically to the three most frequent carcinogenic exposures asbestos, PAH and crystalline silica in an area characterised by its high lung cancer incidence, and a past in heavy industry: iron and coal mining, steel foundries and steel plants. For this, we devised specific exposure questionnaires, which were targeted at being able to get quantitative exposure estimates to the above substances. In this paper, we report on the exposure assessment, and corresponding ORs and AFs based on a case–control study in this area.

## Materials and methods

### Study design

The present study is a population-based lung cancer case–control study in an area consisting of four administrative districts in the Northern part of the French Lorraine region near the German and Luxembourgian borders. This area comprises about 800 000 inhabitants and is characterised by its high lung cancer incidence relative to national data and a past in heavy industry (see online appendix for details on industry and cancer rates). All hospitals located in this area, the hospitals in the nearest major city (Metz) and the reference university hospital in Nancy were contacted and agreed to declare their incident lung cancer cases. Cases were eligible if: (1) they were male and between 40- and 80-year-old, (2) they were resident in the study region, (3) their lung cancer was histologically confirmed and (4) they gave written statements of informed consent. The hospital physician informed the patient of the study and transmitted the patient's address and telephone to the study team if he agreed to participate.

Eight hundred male controls aged between 40 and 80, agreeing to participate, were selected by a random digit dialling procedure in the study area, and were stratified by four 10-year age-classes, the administrative districts and large socioeconomic classes (SEC—5 classes). The strata sizes were obtained by applying age-specific lung cancer incidence rates on the district × SEC × age population proportions.

All cases and controls were contacted by telephone at home and, if they agreed, were interviewed at home and filled in a series of questionnaires in a face-to-face interview lasting usually between 1 and 2 h by trained interviewers with respect to their occupational exposure, smoking, food, beverages, and their personal and familial history of diseases.

The interviews started in February 2006 and finished in December 2010, although not all the hospitals participated during the whole study period. The study was approved by the ethics committee of the national data protection agency (Comité Consultatif sur le traitement de l′information en matière de recherche dans le domaine de la santé) devoted to human studies.

### Exposure assessment

Exposure to smoking was assessed using a detailed questionnaire on all different types of smoking (cigarettes, pipes, cigarillos and cigars). An ever smoker was defined as someone who had smoked at least 100 cigarettes in his lifetime. Smoking was summarised in age at starting, duration of smoking, time since cessation and equivalent pack-years. Diet was assessed with a four-category frequency code of consumption of fresh vegetables and fresh fruits. A lifelong quantification of different beverages (coffee, tea, sodas, wine, beer, cider and spirits) was also obtained.

The occupational exposure was first assessed by obtaining a lifelong list of all jobs held for at least 3 months. These jobs were coded using the ISCO-68 code along with the NAF 2000 (the French coding system of the activities similar to the European NACE coding scheme).

Two series of questionnaires were applied: first questionnaire, for each job a general description of exposures was applied and one or more specific exposure questionnaires (that is, the job-specific questionnaires) were applied if the job title of any given job triggered one or more items of a list of 20 jobs or activities. These questionnaires were developed by one of the authors (JF) for the recent ICARE study (Papadopoulos *et al*, 2011). These specific questionnaires consisted in a comprehensive list of questions with respect to tasks carried out for this job by the worker himself or neighbouring workers, the corresponding daily, weekly or monthly frequencies and the use of individual or collective protection devices.

A second questionnaire, that is, the task-based questionnaire, developed by the corresponding author (CP), consisted 47 task-specific questions. These questions covered the whole job history and were divided, independently for each question, into up to four periods of homogeneous exposures not necessarily consistent with the job periods. Each of these periods was further characterised by a frequency code. The questions covered most of the recogniszed or suspected carcinogens. Thus, exposure to crystalline silica, diesel motor exhaust (DME), stainless steel welding, other welding, other chromium/nickel exposure, iron mining, strong acids, beryllium, cadmium, arsenic, formaldehyde, bis-chloromethyl-ether and radioactivity (the latter consisted in any exposure to radon and radioisotopes) was assessed by one to three questions. Asbestos exposing tasks (15 questions) and tasks involving exposure to PAH-5 questions were assessed in more detail.

The first series of exposure estimates was based on job-specific questionnaires. The authors with the help of several experienced industrial hygienists assigned to each item of the job-specific questionnaires: a semiquantitative exposure level in fibers per ml for asbestos, man-made mineral fibers (MMMF) excluding ceramic fibers, microfibers and other types of fibers and refractory ceramic fibers (RCF) (0: non exposed, 1: <0.1 fibers per ml, 2: 0.1–1 fibers per ml, 3: 1–10 fibers per ml and 4: >10 fibers per ml), for PAH in ng m^−3^ (non exposed, <200 ng m^−3^, 200–1000 ng m^−3^, >1000 ng m^−3^) and crystalline silica in arbitrary units (1, 10 and 100 U). Moreover, a probability code was assigned to each item expressing the hygienists’ certainty in the previous semiquantitative assessment: (1) possible, (2) probable, and (3) certain. These ratings were dependent on calendar period in which the exposure took place, if the hygienists thought that the exposure identified in the respective item had either decreased or, more rarely, increased over time. For each job and each moment, the maximum exposure code for across all items was identified (if the probability code was probable or certain) and the median exposure assigned was multiplied by the duration in years the job was held, thus yielding job-specific cumulative scores. These job-specific scores were then summed up over the whole job history, yielding a cumulative exposure index in years.fibers per ml for asbestos, MMMF and RCF, in years.ng m^−3^ for PAH and in years.u for crystalline silica. Unfortunately, the within-job exposure frequency could not be included in the calculation of these cumulative indices as the different item-specific frequency codes could not be combined as it was not clear whether these items corresponded to simultaneous exposures or not.

The second series of cumulative exposure estimates was based on the task-based questionnaire and was obtained only for asbestos and PAH. Each of the 15 asbestos and the 5-PAH questions were assigned by the authors to one of the same semiquantitative exposure classes as the ones used for the specific questionnaires. The median exposure was multiplied by the assigned exposure frequency and by the duration of exposures coded in these questions yielding a frequency-weighted cumulative exposure index for asbestos in years.fibers per ml and for PAH in years.ng m^−3^.

All these assessments were computer-programmed and were thus independent of the case–control status.

### Statistical analysis

All statistical analyses were conducted using Stata (StataCorp. 2009. *Stata Statistical Software: Release 11*. College Station, TX, USA: StataCorp. LP). The effect of the cumulative exposure indices were modelled using unconditional logistic regression adjusted on the stratifying variables district, age-class and SEC. Moreover, the analyses were adjusted on cumulative smoking by fitting pack-years as a continuous variable (log(pack-year+1)) as well as years since smoking cessation and age as continuous variables. The other potential non-occupational confounding variables (individual history of cancer, family history of lung cancer and flavonoid-containing consumptions, daily fresh fruit, daily fresh vegetable, tea and wine) were all categorical variables and were kept in the model if their significance reached at least a *P*-value of 5%.

For the occupational variables, we first fitted a model (adjusted on the stratification variables, age, cumulative smoking, years since smoking cessation and the non-occupational confounding variables selected in a first step) including cumulative exposure indices for asbestos, PAH, crystalline silica, MMMF and RCF as well as indicator variables for every exposure (based on the task-based questionnaire) for which at least five cases had been found exposed among the following: welding, other chromium/nickel exposure, iron mining, strong acids, beryllium, cadmium, arsenic, formaldehyde, bis-chloromethyl-ether, radioactivity and DME exposure.

The cumulative exposure indices were fitted as continuous variables (log(cumulative exposure+1)). The frequency-weighted indices based on the task-based questionnaire were used for asbestos and PAH whereas for crystalline silica, MMMF and RCF the indices based on the job-specific questionnaire were used for which the exposure-frequency could not be included. From this full exposure models, a backward selection procedure was applied in which the qualitative and cumulative indices were kept in the model if their statistical significance reached at least a *P*-value of 10%. This higher significance level was applied, as these other occupational exposures were considered as potential confounders that needed to be included to assess the effect of the main variables of interest, asbestos, PAH and crystalline silica, which were included in the model regardless of their level of significance. Finally, the joint effect of the main exposures was investigated by fitting the cumulative asbestos exposure index separately in groups based on crystalline silica and PAH.

On the basis of these models, we fitted the corresponding logistic models in which the cumulative variables were replaced by the indicator variables of their quartiles.

For the selected models, AFs and their confidence intervals (CI) were computed using the method of Greenland and Drescher (Greenland and Drescher, 1993) for the logistic model adapted to case–control studies using the procedure aflogit (Brady, 1998). Attributable fractions were computed for each occupational exposure index and globally for all occupational exposures.

## Results

The study comprised 246 lung cancer cases and 531 controls. Participation rates are shown in Supplementary Table S1. Histologies are roughly as expected. Table 1 shows the non-occupational characteristics of the study participants. Neither age-class nor education differed significantly between cases and controls in expected stark contrast to the smoking indices. It is noticeable that the difference in pack-years between cases and controls is mostly due to longer smoking whereas the daily smoking intensity is only slightly higher in cases than in controls. Fresh fruit/vegetable and tea consumption did not differ significantly between cases and controls in contrast to wine consumption. A personal history of cancer and a family history of lung cancer are significantly associated with the case status. However, when modelling simultaneously all these factors adjusted on the stratification variables (Supplementary Table S2) only smoking, an early onset of family lung cancer history, (odds ratios (ORs)=3.27) and a moderate wine consumption (OR=0.54) are statistically significant. The percentages of exposure among both cases and controls (Table 2) are very high (>50%) for crystalline silica as well as asbestos. The exposure prevalences (excluding DME) for PAH are lower (20%) but not negligible. The crude ORs increase with the cumulative exposure indices to all three exposures and exceed three in the highest quartiles. The crude ORs also increase, although less steeply with the cumulative exposure indices to ceramic (RCF) and non-ceramic MMMF (Supplementary Table S3), and are significantly increased with respect to the presence of exposure to stainless steel welding, iron mining and DME (Supplementary Table S4). Adjusted on the other occupational and non-occupational factors, only asbestos, crystalline silica, PAH and DME were significant risk factors. Neither RCF, MMMF, iron mining, stainless steel welding nor any other occupational exposure had any significant relation with lung cancer. Table 3 shows the final model obtained using the backward selection procedure, which includes asbestos, crystalline silica, PAH and DME exposure, and the corresponding estimated AFs. Supplementary Table S5 showing the model adjusted on all potential carcinogens considered shows the same statistical significances than the selected model. Fitting the exposure variables as quartiles shows that the adjusted ORs follow the same increasing pattern than the crude ORs, although the ORs are generally lower than the unadjusted ones. The fraction of the lung cancers attributable to the occupational was >50%, the major contributors to these AFs being asbestos and crystalline silica. Finally, no evidence of a specific effect of concomitant exposures was found (data not shown).

## Discussion

The computed AFs for occupational factors were >50% in our study and were thus higher than most published figures. The most important contributors were the most common recognised carcinogens: asbestos and crystalline silica, PAH as well as DME. Contrary to most entries in the scientific literature, the AFs are reported by carcinogens and based on dose–response relationships with cumulative exposures, exploring quantitatively all the major occupational and non-occupational risk factors simultaneously.

These high AFs correspond to high exposure prevalences that were to some extent expected, as the past in heavy industry was one of the reasons why this area had been chosen in the first place. As the lung cancer mortality exceeds national rates by about 30% (see online appendix), a 50% AF seems consistent with an AF of 20% nationwide (Imbernon, 2003) if one assumes that this excess mortality is only because of occupational factors.

For asbestos, one can compare the observed 52% exposure prevalence among controls to a 54–68% of possibly asbestos-exposed controls in a recent mesothelioma case–control study (Lacourt *et al*, 2010) (although their definition included also possible exposure to asbestos). The high asbestos exposure prevalence is also confirmed by the high pleural mesothelioma rates (see online appendix). Thus, a 20% asbestos-specific AF does appear consistent with a 12% nationwide AF (Imbernon, 2003). In a German population-based case–control study (Pohlabeln *et al*, 2002) in which the control prevalence of asbestos exposure was assessed in a similar way to our study, the asbestos prevalence was 34%. One can note that in the latter study the dose–response slope 1.178 (95% CI 1.052–1.318) is remarkably similar to our 1.182 estimate. The lung cancer risk in relation with asbestos exposure has been confirmed numerous times but in case–control studies, dose–response relationships are still relatively rare.

The carcinogenicity of crystalline silica has been widely discussed even after its classification as a human carcinogen by IARC in 1997 (Soutar *et al*, 2000). Indeed, the dose–response relationship for crystalline silica could only be shown in relatively few studies and mostly in studies published recently: Cassidy *et al* (2007) in a European multicenter study, Vida *et al* (2010) in two Montreal case–control studies and Bruske-Hohlfeld *et al* (2000). All reported significant trends and ORs between 1.5 and 2 in their highest exposure groups similar to ours. However, the percentages of exposed controls varied between 6 and 20% ever-exposed controls according to country and study, well below the >60% exposed in the controls of our study.

For PAH, the results are no less controversial. Armstrong and Gibbs (Armstrong and Gibbs, 2009) found a clear dose–response relationship albeit in an industrial cohort study. Three case–control studies in Canada (Nadon *et al*, 1995), Sweden (Gustavsson *et al*, 2000) and Germany (Bruske-Hohlfeld *et al*, 2000), found significant dose–response relationships with ORs between 1.6 and 2.1 in their respective highest exposure group, which is comparable to our estimate of 1.8. On the other hand, as a result of a large European multicenter case–control, Olsson *et al* (2010) concluded that ‘Occupational PAH exposure does not appear to substantially contribute to the burden of lung cancer in the EU’ based on an 0.93 OR in Central and Eastern Europe, although their sub-study in the United Kingdom showed a significant 1.97 OR, which the authors explained by a possible confounding with asbestos. This UK study with its 17.6% exposure prevalence yields however quite similar results to our study.

Our significant OR for DME provides rather weak evidence, as we could not devise a cumulative exposure estimate. However, our results are again comparable both with respect to the prevalence of exposure and OR to a recent combined analysis of 11 case–control studies from Europe and Canada (Olsson *et al*, 2011), which found an overall 37% prevalence among controls and a summary OR of 1.4 in the highest exposure group.

The exposure to other recognised occupational carcinogens was tested but none reached statistical significance, mostly because the number of exposed subjects was very small. A special case is iron mining, which as an activity is a recognised carcinogen (IARC) and which, in the raw data, is a significant factor, however when adjusted on the stratification variables and the other risk factors is no longer significant. Iron mining occurs mostly in one of the administrative districts, which are part of the stratification. Adjustment on these districts may have led to over adjustment. An alternative explanation might be that iron mining is not a risk factor *per se* and that when the concomitant risk factors (DME, crystalline silica, and so on) are taken into account, iron mining is no longer a risk factor. The unadjusted ORs for exposure to MMMF were increased but when adjusted on asbestos no risk at all was discernible, which confirms a conclusion reached by Lipworth *et al* (2009) who argue against a carcinogenic effect of MMMF because, among other reasons, the observed risks in many studies are explainable by residual confounding by smoking and asbestos. A similar finding is observed for stainless steel welding, whose unadjusted OR is significantly increased, but whose adjusted OR is close to unity.

These results must be put in the context of the strengths and weaknesses of our study. Our study has some obvious weaknesses with a relatively small number of cases, which is partially due to a less than optimal response rate in cases. To some extent, this response rate is because of the fact that the cases were interviewed at home rather than in the hospital that however makes them more comparable to the controls, as the data collection was done in the same circumstances. This small number of cases reduced the power of the study but the fact that the target exposures showed significant dose effects is proof enough that the study size was large enough. Another important power determinant when estimating dose–response effects is the exposure variance. The latter was quite high, because of the choice of a population with a past of heavy industry so that the small number of cases was counterbalanced by this large exposure variance. A further possible weakness is the control selection process based on random digit dialling. Although this method is probably soon obsolete, one can stress that on one hand most people aged between 40 and 80 (median 64) still have fixed telephone lines and, on the other hand, that the few cases who could not be reached by telephone were not included either. The age range in our study is quite wide, but restricting the analysis to age >55 years did not change the results (data not shown). The hospitals’ participation in the case ascertainment was unequal but we have no reason to suspect that this resulted in any bias and the possible effect was partially controlled for by stratifying on the administrative district. Although the study area was home to heavy industries (see online appendix), we are not aware of major environmental hazards and given that cases and controls are frequency matched on residential district, we do not think that the occupational ORs could have been confounded to any degree by environmental exposures.

Strengths of the current study include the fact that data were collected by a small number of experienced interviewers and that the questionnaires were computer-coded by two interviewers in the weeks after the interview, and the questionnaires could therefore be quality-checked by technicians knowing both the context and the content of the study. This system allowed a feedback to all interviewers with respect to the homogeneity of interviews. The major strength of our study is, however, the detailed exposure descriptions that were obtained and the quantitative indices of cumulative exposure that have been derived from them and that have allowed in the first place to be able to obtain these dose–response relationships, although exposure-frequency could not be included in the index for crystalline silica, which might have attenuated the dose–effect relationship. The assessment of the job history was quite detailed and indeed, 18% of the jobs coded with the highest asbestos code occurred in jobs held for <1 year. This detailed quantitative exposure assessment was recently shown to be associated with steeper dose–response slopes (Lenters *et al*, 2011) and might have also increased the statistical power. A formal comparison of the performance of our algorithms with other metrics based on expert assessments and JEMs is under way but out of the scope of this paper. Shortly, the exposure metrics used in this paper proved both sensitive and specific for asbestos but less so for PAH, compared with a gold standard based on a detailed expert assessment based on all available data.

Moreover, the detailed questionnaires covered not only the occupational factors but allowed us to assess simultaneously several other factors. Thus, we could show the increasing benefit of time since cessation of smoking and explore the effect of personal and familial cancer risk. Interestingly and contrary to the findings of Cassidy *et al* (2008), the significant OR for personal cancer history disappeared after adjustment on the other confounders, suggesting that these prior cancer diagnoses had common risk factors with the present lung cancer most likely smoking and/or occupational factors. It is to be stressed that the lung cases included in our study were all primary cancers. On the contrary, when we explored the family of lung cancer risk by age of onset we found, similarly to Cassidy *et al* (2009), that an early age of onset was related to the lung cancer risk, suggesting that early onset lung cancer may have a genetic component. When trying to duplicate the findings of Cui *et al* (2008) with respect to protective effects of flavonoid dietary intake, the present study was less successful, possibly because our questionnaire was not specifically targeted on nutritional outcomes. We did not find any benefit of fresh fruit or fresh vegetable consumption. This might be also because of a residual confounding as (data not shown) we found that fresh fruit and fresh vegetable consumption increased strongly with age. Finally, the single flavonoid containing consumption that showed a significant protective effect, is a moderate wine consumption. The data are too scarce to interpret this finding causally, and it could well be that a moderate wine consumption (>80% of the controls are at least occasional wine drinkers) is a marker of an overall moderate lifestyle. Thus, when adjusting on wine consumption, we also to some extent adjust on lifestyle.

A last aspect to be discussed is the issue of compensation. In the online appendix, we show that about 10% of the lung cancer cases of the study area are recognised to be due to occupational factors. This is much lower than the 50% AF. However, in order to be compensated, an individual case must have been highly exposed whereas AFs include also the (population) effects of lower levels of exposure. Moreover, the French insurance system does not recognise cases exposed to crystalline silica or to DME as eligible for compensation except in very specific circumstances.

In summary, our study did not only model jointly all the major occupational risk factors (with cumulative exposure indices), but also in addition a detailed assessment of smoking and some major non-occupational risk and protective factors giving a comprehensive view of risk factors of lung cancer.

As a conclusion, our results show that, at least in areas with hazardous industries, occupational lung cancers are by no means a thing of the past and the preventable fraction of lung cancers is non-negligible. A second conclusion is that if the lung cancers due to asbestos-exposure will eventually fade out following banishment, this is not the case for the other carcinogens crystalline silica, PAH and DME, for which the effort for prevention must be sustained. Moreover, studying interactions between these factors, and relationships with the main lung cancer phenotypes (Paris *et al*, 2010), may be of interest too in next future to better understand, treat or prevent this disease.

Finally, in a time in which the secondary prevention of lung cancer by computed chest tomography of high-risk subjects is increasingly considered to be useful (Aberle *et al*, 2011), these figures highlight the need to include occupational risk factors in addition to smoking in prediction models used to identify high-risk subjects. For this, it is important to develop simplified questionnaires that can be applied out of the context of a scientific study. Our task-based questionnaires are a first step in this direction.

## Figures and Tables

**Table 1:** Non-occupational characteristics of lung cancer cases and controls

	**Controls (*n*=531)**		**Cases (*n*=246)**		
**Variable**	**No.**	**% or IQR**	**No.**	**% or IQR**	***P*-value**
*Age class*					0.27
40–49	53	10.0%	14	5.7%	
50–59	140	26.4%	67	27.2%	
60–69	177	33.3%	86	35.0%	
70–79	161	30.3%	79	32.1%	
					
*Highest education level (years of schooling)*					0.23
Upper primary school (7 years)	84	15.8%	45	18.3%	
Mid-secondary school (9 years)	20	3.8%	13	5.3%	
Low-level technical diploma (11 years)	240	45.2%	112	45.5%	
Upper education (>12 years)	97	18.3%	30	12.1%	
Other or missing	90	17.0%	46	18.7%	
					
*Smoking*					<0.0005
Lifelong non-smoker	121	22.8%	8	3.3%	
Ever smoker	410	77.2%	238	96.8%	
Median duration in years of smoking (IQR) among smokers	29	(20–38)	39.5	(32–46)	<0.0005
Median pack-years (IQR) among smokers	25.5	(12.6–39.5)	36.8	(25.3–50.6)	<0.0005
					
*Approximate quartiles of pack-years*					
Q1	121	22.8%	22	18.4%	
Q2	119	22.4%	59	22.9%	
Q3	95	17.9%	70	21.2%	
Q4	75	14.1%	87	20.9%	<0.0005
Median g tobacco per day (IQR) among smokers	18.5	(12.3–22.2)	′19.5	(15.0–25.9)	0.02
					
*Flavonoid-containing consumptions*					
Fresh fruit once a day	303	57.1%	155	63.0%	0.12
Fresh vegetables once a day	221	41.6%	111	45.1%	0.35
Daily tea	65	12.2%	23	9.4%	0.22
Wine consumption					0.001
Never	86	16.2%	56	22.6%	
Less than daily	151	28.4%	38	15.5%	
Daily	294	55.4%	152	61.8%	
					
Personal cancer history	65	12.2%	48	19.5%	0.007
					
*Familial lung cancer history*					0.02
No	497	93.6%	218	88.6%	
Early onset (<60 years)	8	1.5%	11	4.5%	
Late onset (⩾60 years)	26	4.9%	17	6.9%	

Abbreviation: IQR=inter quartile range.

**Table 2:** Exposure of cases and controls according to task-based (Asbestos and PAH) and job-specific (crystalline silica) scores

	**Asbestos**			**PAH**			**Crystalline silica**		
	**Controls (%)**	**Cases (%)**	**OR**	**Controls (%)**	**Cases (%)**	**OR**	**Controls (%)**	**Cases (%)**	**OR**
*Maximum level* a									
Non-exposed	255 (48.0)	71 (28.9)	1.0	408 (76.8)	154 (62.6)	1.0	194 (36.5)	53 (21.5)	1.0
Unknown but self-reported	18 (3.4)	13 (5.3)	2.6^*^						
L1	105 (19.8)	40 (16.3)	1.4	83 (15.6)	57 (23.2)	1.8^**^	98 (18.3)	32 (13.0)	1.2
L2	119 (22.4)	77 (31.3)	2.3^***^	5 (1)	1 (0.4)	0.5	130 (24.5)	73 (29.7)	2.1^***^
L3	34 (6.4)	45 (18.3)	4.8^***^	35 (6.6)	34 (13.8)	2.6^***^	109 (20.5)	88 (35.8)	3.0^***^
									
*Cumulative exposure by approximate quartiles*									
Non-exposed	255 (48.0)	71 (28.9)	1.0	408 (76.8)	154 (62.6)	1.0	194 (36.5)	53 (21.5)	1.0
Q1	80 (15.1)	34 (13.8)	1.5	45 (8.5)	19 (7.7)	1.1	97 (18.3)	32 (13.0)	1.2
Q2	72 (13.6)	40 (16.3)	2.0^*^	35 (6.6)	29 (11.8)	2.2^**^	85 (16.0)	46 (18.7)	2.0^**^
Q3	72 (13.6)	46 (18.7)	2.3^***^	24 (4.5)	21 (8.5)	2.3^**^	81 (15.3)	53 (21.5)	2.4^***^
Q4	52 (9.8)	55 (22.4)	3.8^***^	19 (3.6)	23 (9.4)	3.2^***^	74 (13.9)	62 (25.2)	3.1^***^

Abbreviations: OR=odds ratios; PAH=polycyclic aromatic hydrocarbons.

^*^P<0.05, ^**^P<0.01 ^***^P<0.001.

a Asbestos: L1= <1 fiber per ml, L2= 1–10 fibers per ml, L3=>10fiber per ml; PAH: <200 ng m^−3^, 200–1000 ng m^−3^, >1000 ng m^−3^; Crystalline silica=1, 10, 100 arbitrary units.

**Table 3:** Selected multiple logistic model and corresponding attributable risks^a^

	**Continuous exposure variables**		**Exposure variables in quartiles**	
	**OR**	**95% CI**	**OR**	**95% CI**
*Smoking*				
Ln (packyears +1)	1.97	1.67, 2.33		
Non-smokers			1	
Q1			6.6	2.5, 17.9
Q2			15.1	6.2, 36.7
Q3			15.4	6.5, 36.1
Q4			23.0	9.9, 53.6
Years since smoking cessation	0.964	0.948, 0.980	0.955	1.027, 1.174
				
Age (years)	1.091	1.023, 1.165	1.098	
				
*Wine consumption*				
Never drinker	1		1	
Less than daily	0.57	0.32–1.01	0.55	0.31, 0.99
Daily	0.75	0.47, 1.20	0.75	0.47, 1.22
				
*Family history of lung cancer*				
No history	1		1	
Early onset (<60 years)	4.01	1.23, 13.03	4.01	1.20, 13.40
Late onset (>60 years)	1.37	0.66, 2.84	1.35	0.64, 2.83
				
**Exposure to DME**	**AF=15% (3, 25%)**		**AF=14% (2, 25%)**	
	1.66	1.11, 2.49	1.62	1.07, 2.44
				
**Asbestos**	**AF** **=22% (9, 34%)**		**AF=31% (10, 47%)**	
Ln (years fibers per ml +1)	1.182	1.064, 1.313		
Non-exposed			1	
Q1			1.30	0.73, 2.30
Q2			1.60	0.90, 2.83
Q3			1.67	0.94, 2.94
Q4			2.70	1.50, 4.87
				
**PAH**	**AF=11% (2, 20%)**		**AF=8% (−7, 21%)**	
Ln (years ng m^−3^ +1)	1.166	1.036, 1.312		
Non-exposed			1	
Q1			0.66	0.33, 1.31
Q2			1.32	0.68, 2.59
Q3			2.08	0.96, 4.47
Q4			2.02	0.92, 4.45
				
**Crystalline silica**	**AF=30% (6, 49%)**		**AF=17% (−17, 42%)**	
Ln (years u +1)	1.07	1.011, 1.140		
Non-exposed			1	
Q1			0.88	0.48, 1.60
Q2			1.16	0.64, 2.13
Q3			1.36	0.76, 2.44
Q4			1.76	0.96, 3.21
				
**Overall attributable fraction to occupational factors**	**AF=56% (41, 67%)**		**AF=52% (32, 66%)**	

Abbreviations: AF=attributable fraction; CI=confidence intervals; DME=diesel motor exhaust; OR=odds ratios; PAH=polycyclic aromatic hydrocarbons.

a Adjusted on stratification variables.

## References

[bib1] Aberle DR, Adams AM, Berg CD, Black WC, Clapp JD, Fagerstrom RM, Gareen IF, Gatsonis C, Marcus PM, Sicks JD (2011) Reduced lung-cancer mortality with low-dose computed tomographic screening. N Engl J Med 365(5): 395–409 21714641 10.1056/NEJMoa1102873 PMC4356534

[bib2] Armstrong BG, Gibbs G (2009) Exposure-response relationship between lung cancer and polycyclic aromatic hydrocarbons (PAHs). Occup Environ Med 66: 740–746 19546103 10.1136/oem.2008.043711

[bib3] Blot WJ, Fraumeni JF (1996) Cancers of the lung and pleura. In: Cancer Epidemiol Prev, Schottenfeld DJF (ed). pp 637–665. Oxford University Press: New-York

[bib4] Brady A (1998) sbe21-Adjusted population attributable fractions from logistic regression. Stata Technical Bulletin 42: 8–12

[bib5] Bruske-Hohlfeld I, Mohner M, Pohlabeln H, Ahrens W, Bolm-Audorff U, Kreienbrock L, Kreuzer M, Jahn I, Wichmann HE, Jockel KH (2000) Occupational lung cancer risk for men in Germany: results from a pooled case-control study. Am J Epidemiol 151: 384–395 10695597 10.1093/oxfordjournals.aje.a010218

[bib6] Cassidy A, Balsan J, Vesin A, Wu X, Liloglou T, Brambilla C, Timsit JF, Field JK (2009) Cancer diagnosis in first-degree relatives and non-small cell lung cancer risk: results from a multi-centre case-control study in Europe. Eur J Cancer 45: 3047–3053 19482469 10.1016/j.ejca.2009.05.006

[bib7] Cassidy A, Myles JP, van Tongeren M, Page RD, Liloglou T, Duffy SW, Field JK (2008) The LLP risk model: an individual risk prediction model for lung cancer. Br J Cancer 98: 270–276 18087271 10.1038/sj.bjc.6604158 PMC2361453

[bib8] Cassidy A, t Mannetje A, van Tongeren M, Field JK, Zaridze D, Szeszenia-Dabrowska N, Rudnai P, Lissowska J, Fabianova E, Mates D, Bencko V, Foretova L, Janout V, Fevotte J, Fletcher T, Brennan P, Boffetta P (2007) Occupational exposure to crystalline silica and risk of lung cancer: a multicenter case-control study in Europe. Epidemiology 18: 36–43 17149143 10.1097/01.ede.0000248515.28903.3c

[bib9] Cui Y, Morgenstern H, Greenland S, Tashkin DP, Mao JT, Cai L, Cozen W, Mack TM, Lu QY, Zhang ZF (2008) Dietary flavonoid intake and lung cancer--a population-based case-control study. Cancer 112: 2241–2248 18327817 10.1002/cncr.23398 PMC5546301

[bib10] Damber L, Larsson LG (1985) Underground mining, smoking, and lung cancer: a case-control study in the iron ore municipalities in northern Sweden. J Natl Cancer Inst 74: 1207–1213 3858594

[bib11] De Matteis S, Consonni D, Bertazzi PA (2008) Exposure to occupational carcinogens and lung cancer risk. Evolution of epidemiological estimates of attributable fraction. Acta Biomed 79(Suppl 1): 34–42 18924308

[bib12] Greenland S, Drescher K (1993) Maximum likelihood estimation of the attributable fraction from logistic models. Biometrics 49: 865–872 8241375

[bib13] Gustavsson P, Jakobsson R, Nyberg F, Pershagen G, Jarup L, Scheele P (2000) Occupational exposure and lung cancer risk: a population-based case-referent study in Sweden. Am J Epidemiol 152: 32–40 10901327 10.1093/aje/152.1.32

[bib14] Imbernon E (2003) Estimation du Nombre De Cas De Certains Cancers Attribuables À Des Facteurs Professionnels En France. Insititut de Veille Sanitaire: Paris France. 1–28

[bib15] Lacourt A, Rolland P, Gramond C, Astoul P, Chamming's S, Ducamp S, Frenay C, Galateau-Salle F, Gilg Soit Ilg A, Imbernon E, Le Stang N, Pairon JC, Goldberg M, Iwatsubo Y, Salmi LR, Brochard P (2010) Attributable risk in men in two French case-control studies on mesothelioma and asbestos. Eur J Epidemiol 25: 799–806 20821038 10.1007/s10654-010-9502-0

[bib16] Lenters V, Vermeulen R, Dogger S, Stayner L, Portengen L, Burdorf A, Heederik D (2011) A meta-analysis of asbestos and lung cancer: is better quality exposure assessment associated with steeper slopes of the exposure-response relationships? Environ Health Perspect 119: 1547–1555 21708512 10.1289/ehp.1002879 PMC3226488

[bib17] Lipworth L, La Vecchia C, Bosetti C, McLaughlin JK (2009) Occupational exposure to rock wool and glass wool and risk of cancers of the lung and the head and neck: a systematic review and meta-analysis. J Occup Environ Med 51: 1075–1087 19730396 10.1097/JOM.0b013e3181b35125

[bib18] Nadon L, Siemiatycki J, Dewar R, Krewski D, Gerin M (1995) Cancer risk due to occupational exposure to polycyclic aromatic hydrocarbons. Am J Ind Med 28: 303–324 7485186 10.1002/ajim.4700280302

[bib19] Olsson AC, Fevotte J, Fletcher T, Cassidy A, t Mannetje A, Zaridze D, Szeszenia-Dabrowska N, Rudnai P, Lissowska J, Fabianova E, Mates D, Bencko V, Foretova L, Janout V, Brennan P, Boffetta P (2010) Occupational exposure to polycyclic aromatic hydrocarbons and lung cancer risk: a multicenter study in Europe. Occup Environ Med 67: 98–103 19773276 10.1136/oem.2009.046680

[bib20] Olsson AC, Gustavsson P, Kromhout H, Peters S, Vermeulen R, Brüske I, Pesch B, Siemiatycki J, Pintos J, Brüning T, Cassidy A, Wichmann HE, Consonni D, Landi MT, Caporaso N, Plato N, Merletti F, Mirabelli D, Richiardi L, Jöckel KH, Ahrens W, Pohlabeln H, Lissowska J, Szeszenia-Dabrowska N, Zaridze D, Stücker I, Benhamou S, Bencko V, Foretova L, Janout V, Rudnai P, Fabianova E, Dumitru RS, Gross IM, Kendzia B, Forastiere F, Bueno-de-Mesquita B, Brennan P, Boffetta P, Straif K (2011) Exposure to diesel motor exhaust and lung cancer risk in a pooled analysis from case-control studies in Europe and Canada. Am J Respir Crit Care Med 183: 941–948 21037020 10.1164/rccm.201006-0940OC PMC7259806

[bib21] Papadopoulos A, Guida F, Cenee S, Cyr D, Schmaus A, Radoi L, Paget-Bailly S, Carton M, Tarnaud C, Menvielle G, Delafosse P, Molinie F, Luce D, Stucker I (2011) Cigarette smoking and lung cancer in women: results of the French ICARE case-control study. Lung Cancer 74(3): 369–377 21620510 10.1016/j.lungcan.2011.04.013

[bib22] Paris C, Clement-Duchene C, Vignaud JM, Gislard A, Stoufflet A, Bertrand O, Thiberville L, Grosdidier G, Martinet Y, Benichou J, Hainaut P (2010) Relationships between lung adenocarcinoma and gender, age, smoking and occupational risk factors: a case-case study. Lung Cancer 68: 146–153 19586681 10.1016/j.lungcan.2009.06.007

[bib23] Pohlabeln H, Boffetta P, Ahrens W, Merletti F, Agudo A, Benhamou E, Benhamou S, Brüske-Hohlfeld I, Ferro G, Fortes C, Kreuzer M, Mendes A, Nyberg F, Pershagen G, Saracci R, Schmid G, Siemiatycki J, Simonato L, Whitley E, Wichmann HE, Winck C, Zambon P, Jöckel KH (2000) Occupational risks for lung cancer among nonsmokers. Epidemiology 11: 532–538 10955405 10.1097/00001648-200009000-00008

[bib24] Pohlabeln H, Wild P, Schill W, Ahrens W, Jahn I, Bolm-Audorff U, Jockel KH (2002) Asbestos fibreyears and lung cancer: a two phase case-control study with expert exposure assessment. Occup Environ Med 59: 410–414 12040118 10.1136/oem.59.6.410 PMC1740301

[bib25] Rushton L, Bagga S, Bevan R, Brown TP, Cherrie JW, Holmes P, Fortunato L, Slack R, Van Tongeren M, Young C, Hutchings SJ (2010) Occupation and cancer in Britain. Br J Cancer 102: 1428–1437 20424618 10.1038/sj.bjc.6605637 PMC2865752

[bib26] Soutar CA, Robertson A, Miller BG, Searl A, Bignon J (2000) Epidemiological evidence on the carcinogenicity of silica: factors in scientific judgement. Ann Occup Hyg 44: 3–14 10689755 10.1016/s0003-4878(99)00047-2

[bib27] Steenland K, Burnett C, Lalich N, Ward E, Hurrell J (2003) Dying for work: the magnitude of US mortality from selected causes of death associated with occupation. Am J Ind Med 43: 461–482 12704620 10.1002/ajim.10216

[bib28] Sun S, Schiller JH, Gazdar AF (2007) Lung cancer in never smokers—a different disease. Nat Rev Cancer 7: 778–790 17882278 10.1038/nrc2190

[bib29] Vida S, Pintos J, Parent ME, Lavoue J, Siemiatycki J (2010) Occupational exposure to silica and lung cancer: pooled analysis of two case-control studies in Montreal, Canada. Cancer Epidemiol Biomarkers Prev 19: 1602–1611 20501770 10.1158/1055-9965.EPI-10-0015

