# Supplementary material for: Occupational risk factors have to be considered in the definition of high-risk lung cancer populations
Source: Br J Cancer. 2012 Mar 27;106(7):1346–52. doi: 10.1038/bjc.2012.75 (PMC3314791; doi:10.1038/bjc.2012.75)
Supplement: Supplementary Tables S1-S5 [file bjc201275x1.doc]

Online Table 1 Participation rates of lung cancer cases according to age and histology

|  | **Decease or too ill to be interviewed** | **Refused to be interviewed or impossible to contact** | **Interviewed** | **Total** |
| --- | --- | --- | --- | --- |
| **Age ^#^** |  |  |  | **P=0.35** |
| **40-49** | 10 (9.8%) | 10 (4.8%) | 14 (5.7%) | 34 (6.1%) |
| **50-59** | 24 (23.5%) | 52 (24.8%) | 67 (27.2%) | 143 (25.6%) |
| **60-69** | 27 (26.5%) | 70 (33.3%) | 86 (35.0%) | 183 (32.8%) |
| **70-79** | 41 (40.2%) | 78 (37.1%) | 79 (32.1%) | 198 (35.5%) |
| **Histology** |  |  |  | **P=0.02** |
| **Epidermoid** | 21 (27.3%) | 53 (32.9%) | 98 (39.8%) | 172 (35.5%) |
| **Adenocarcinoma** | 26 (33.8%) | 50 (31.1%) | 94 (38.2%) | 170 (35.1%) |
| **Small Cell** | 14 (18.2%) | 22 (13.7%) | 24 (9.8%) | 60 (12.4%) |
| **Large Cell** | 1 (1.3%) | 5 (3.1%) | 6 (2.4%) | 12 (2.5%) |
| **Other** | 15 (19.5%) | 31 (19.3%) | 24 (9.8%) | 70 (14.5%) |
| **missing** | 35 | 60 | - | 95 |
| **Total** | **112** | **221** | **246** | **579** |

**^#^** Among the cases who could not be interviewed age was missing for 21 cases

Online Table 2: Multiple logistic modelling of potentially confounding variables ^#^

|  | **Odds ratio** | **P>|z|** | **[95% Conf. Interval]** | |
| --- | --- | --- | --- | --- |
| **Smoking** |  |  |  |  |
| **Ln(packyears +1)** | 1.92 | 0.000 | 1.63 | 2.25 |
| **Time since smoking cessation (years)** | 0.963 | 0.000 | 0.947 | 0.979 |
| **Age (years)** | 1.08 | 0.02 | 1.011 | 1.146 |
| **Daily tea consumption vs. less than daily** | 1.01 | >0.50 | 0.56 | 1.82 |
| **Wine consumption vs. none** |  |  |  |  |
| **Less than daily** | 0.54 | 0.034 | 0.31 | 0.95 |
| **daily** | 0.76 | 0.240 | 0.48 | 1.2 |
| **Daily fresh fruit consumption vs. less than daily** | 1.43 | 0.062 | 0.98 | 2.08 |
| **Daily fresh vegetable consumption vs. less than daily** | 1.23 | 0.329 | 0.83 | 1.74 |
| **Personal history of cancer (yes vs. no)** | 1.43 | 0.140 | 0.89 | 2.31 |
| **Family history of lung cancer vs. no history** |  |  |  |  |
| **Early Onset (<60 years)** | 3.27 | 0.042 | 1.04 | 10.26 |
| **Late Onset (>60 years)** | 1.3 | 0.473 | 0.64 | 2.65 |

^#^ Adjusted on stratification variables

Online Table 3 Exposure of cases and controls to non-ceramic and ceramic man-made mineral fibres based on job-specific questionnaires

|  | **Non-ceramic MMMF** | | | **Ceramic MMMF** | | |
| --- | --- | --- | --- | --- | --- | --- |
|  | **Controls** | **Cases** | **OR** | **Controls** | **Cases** | **OR** |
| **Maximum level** |  |  |  |  |  |  |
| **Non-exposed** | 342 (64.4%) | 134 (54.5%) | **1.0** | 395 (74.4%) | 165 (67.1%) | **1.0** |
| **<1 f/mL** | 42 (6.4%) | 20 (5.7%) | **1.1** | 67 (11.7%) | 35 (12.2%) | **1.2** |
| **1-10f/mL** | 100 (18.8%) | 49 (19.9%) | **1.3** | 47 (8.9%) | 22 (8.9%) | **1.1** |
| **>10f/mL** | 47 (8.9%) | 43 (17.5%) | **2.3***** | 22 (4.1%) | 24 (9.8%) | **2.6**** |
| **Cumulative**  **exposure** |  |  |  |  |  |  |
| **Non-exposed** | 342 (64.4%) | 134 (54.5%) | **1.0** | 395 (74.4%) | 165 (67.1%) | **1.0** |
| **Q1** | 51 (9.6%) | 23 (9.4%) | **1.2** | 35 (6.6%) | 20 (8.1%) | **1.4** |
| **Q2** | 53 (10.0%) | 22 (8.9%) | **1.1** | 36 (6.8%) | 17 (6.9%) | **1.1** |
| **Q3** | 46 (8.7%) | 30 (12.2%) | **1.7*** | 35 (6.6%) | 20 (8.1%) | **1.4** |
| **Q4** | 39 (7.3%) | 37 (15.0%) | **2.4***** | 30 (5.7%) | 24 (9.8%) | **1.9*** |

* p<0.05, ** p<0.01 ***p<0.001

Online Table 4: Exposure to other potential carcinogens based on task questionnaires, with at least 5 exposed cases

|  | **Controls** | **Cases** | **OR** |
| --- | --- | --- | --- |
| **Chromium/Nickel** |  |  |  |
| **Non-exposed** | 481 (91.7%) | 205 (83.3%) | **1.0** |
| **Stainless steel welding** | 41 (7.7%) | 36 (14.6%) | **2.1**** |
| **Other Cr/Ni exposure** | 9 (1.7%) | 5 (2.0%) | **1.8** |
| **Welding** |  |  |  |
| **Non-exposed** | 357 (67.2%) | 169 (68.7%) | **1.0** |
| **Stainless steel welding** | 41 (7.7%) | 36 (14.6%) | **2.0**** |
| **Other welding** | 133 (25.1%) | 54 (22.0%) | **0.9** |
| **Iron mining (ever)** | 33 (6.2%) | 33 (13.4%) | **2.3**** |
| **Strong acids exposure (ever)** | 57 (10.7%) | 28 (11.4%) | **1.1** |
| **Formaldehyde (ever)** | 4 (0.8%) | 5 (2.0%) | **2.7** |
| **Radioactivity (ever)** | 31 (5.8%) | 24 (9.8%) | **1.7*** |
| **DME exhaust** |  |  |  |
| **Non-exposed** | 410 (77.2%) | 155 (63.0%) | **1.0** |
| **Less than 20 years** | 55 (10.4%) | 44 (17.9%) | **2.1**** |
| **More than 20 years** | 66 (12.4%) | 47 (19.1%) | **1.9**** |

* p<0.05, ** p<0.01 ***p<0.001

Online table 5: Multiple logistic model with selected confounders and all occupational exposure variables with at least 5 exposed cases^#^

|  | **Odds ratio** | **P** | **[95% Conf. Interval]** | |
| --- | --- | --- | --- | --- |
| **Smoking** |  |  |  |  |
| **Ln(packyears +1)** | 1.98 | 0.000 | 1.68 | 2.36 |
| **Time since smoking cessation (years)** | 0.96 | 0.000 | 0.95 | 0.980 |
| **Age (years)** | 1.1 | 0.01 | 1.03 | 1.17 |
| **Wine consumption** |  |  |  |  |
| **Less than daily** | 0.58 | 0.065 | 0.32 | 1.04 |
| **Daily** | 0.76 | 0.261 | 0.47 | 1.23 |
| **Family history of lung cancer vs. no history** | |  |  |  |
| **Early Onset (<60 years)** | 3.94 | 0.025 | 1.19 | 13.06 |
| **Late Onset (>60 years)** | 1.4 | 0.370 | 0.67 | 2.91 |
| **Iron mining** | 1.36 | 0.397 | 0.67 | 2.77 |
| **Stainless steel welding** | 1.08 | >0.500 | 0.59 | 1.99 |
| **DME exposure** | 1.46 | 0.033 | 1.04 | 2.4 |
| **Exposure to radioactivity** | 1.51 | 0.263 | 0.73 | 3.1 |
| **Exposure to formaldehyde** | 2.70 | 0.244 | 0.51 | 14.4 |
| **Cumulative asbestos - log( years.f/mL +1)** | 1.16 | 0.020 | 1.02 | 1.31 |
| **Cumulative PAH - log( years.ng/m^3^ +1)** | 1.17 | 0.011 | 1.04 | 1.32 |
| **Cumulative crystalline silica - log( years.u +1)** | 1.07 | 0.04 | 1.00 | 1.14 |
| **Cumulative MMMF- log( years.f/mL +1)** | 1.02 | >0.500 | 0.91 | 1.14 |
| **Cumulative RCF- log( years.f/mL +1)** | 0.99 | >0.500 | 0.86 | 1.14 |

^#^ Adjusted on stratification variables
