# Supplementary material for: Occupational risk factors have to be considered in the definition of high-risk lung cancer populations
Source: Br J Cancer. 2012 Mar 27;106(7):1346–52. doi: 10.1038/bjc.2012.75 (PMC3314791; doi:10.1038/bjc.2012.75)
Supplement: Supplementary Appendix [file bjc201275x2.doc]

Online Appendix: The study region

The study region is an area consisting of four administrative districts in the Northern part of the French Lorraine region near the German and Luxembourgian borders. The study area is part of a larger region consisting of the two “departments” Moselle and Meurthe-et-Moselle, which we’ll denote by MMM. The study area comprises about 800,000 inhabitants and is characterized by its high lung cancer mortality relative to national data and a past in heavy industry. The target population of men aged 40 to 79 consisted of about 170,000 subjects. The MMM area comprises about 1700000 inhabitants and is much less industrialized.

**Industrial history**

This study area is a historical mining region both for iron and coal.

Over 60 iron pits were in activity in the 1940s but the activity gradually declined and most of the pits were closed in the 1970s: in 1962 over 22000 iron miners were still employed, but this number decreased to 10000 in 1972. The last pit was shut down in 1997.

Fifty-eight coal pits were opened between 1818 and 1987 and the last pit closed in 2004. While this industry employed over 43000 miners in the 1960s, only 24000 miners were still employed in the 1980s.

The main industry of the study area was the steel industry in the largest sense with coke production (the last coke ovens are still active), foundries and steel plants. This industry was massively downsized in the 1980s but still employed over 20000 workers in 2008.

The only other large industry is a petrochemical complex set up in the 1950s, which employed several thousand workers in the past but less than 1000 nowadays.

No other large industries (e.g. cement or asbestos factories) potentially exposing to high levels of carcinogens existed in the area.

**Lung cancer in the study area**

The lung cancer mortality rates between 2005 and 2009 for men in the age-groups considered (from 40 to 79) show an excess compared to France which increases with age (see figure 1)

Figure 1: Lung cancer mortality rates in France and the study region

Overall the lung cancer SMR of the study region compared to France is 1.27.

**Lung cancer and pleural mesothelioma in the MMM area**

Neither the number of compensated lung cancer cases nor the pleural mesothelioma rates were available at the study area level so that we give these figures in the larger MMM area.

*Compensated lung cancer cases*

Between 2005 and 2008, 243 lung cancer cases in males between 40 and 79 were compensated in the MMM area. This is to be compared to 2561 deaths from lung cancer in the same age classes and the same area.

*Pleural mesothelioma*

In the MMM area, the pleural mesothelioma mortality rates between 2005 and 2009 for men in the age-groups considered (from 40 to 79) show an excess compared to France which increases with age (see figure 2).

Figure 2: Pleural mesothelioma mortality rates in France and the MMM area

Overall the pleural mesothelioma SMR of the MMM area compared to France is 1.54.

Given the differences in the socio-economic structure between the study area and MMM area in which it is included, and hence the differences in exposure, it is likely that the pleural mesothelioma SMR in the study region is still higher than in the MMM area.
